# Delayed concentration effect models for dabigatran anticoagulation

**DOI:** 10.1111/pan.14511

**Published:** 2022-07-02

**Authors:** Michael P. Eaton, Sergiy M. Nadtochiy, Tatsiana Stefanos, Dana LeMoine, Brian J. Anderson

**Affiliations:** ^1^ University of Rochester School of Medicine and Dentistry Rochester New York USA; ^2^ Department Anesthesiology University of Auckland Auckland New Zealand

**Keywords:** cardiopulmonary bypass, coagulation, dabigatran, pharmacokinetics, pharmacokinetics

## Abstract

**Introduction:**

Dabigatran is an anticoagulant with potential use during cardiopulmonary bypass in children and adults. The pharmacokinetic–pharmacodynamic relationship for dabigatran anticoagulation effect was investigated in an intact animal model using rabbits.

**Methods:**

Ten male New Zealand white rabbits were given a novel preparation of intravenous dabigatran 15 mg.kg^−1^. Blood samples were collected for activated clotting time, thromboelastometric reaction time, and drug assay at 5, 15, 30, 60, 120, 180, 300, and 420 min. Plasma dabigatran concentrations and coagulation measures were analyzed using an integrated pharmacokinetic–pharmacodynamic model using nonlinear mixed effects. Effects (activated clotting and thromboelastometric reaction times) were described using a sigmoidal E_MAX_ model. Pharmacokinetic parameters were scaled using allometry and standardized to a 70 kg size standard. Pharmacodynamics were investigated using both an effect compartment model and an indirect response (turnover) model.

**Results:**

A two‐compartment model described dabigatran pharmacokinetics with a clearance (CL 0.135 L.min^−1^.70 kg^−1^), intercompartment clearance (Q 0.33 L.min^−1^.70 kg^−1^), central volume of distribution (V1 12.3 L.70 kg^−1^), and peripheral volume of distribution (V2 30.1 L.70 kg^−1^). The effect compartment model estimates for a sigmoid E_MAX_ model with activated clotting time had an effect site concentration (Ce_50_ 20.1 mg.L^−1^) eliciting half of the maximal effect (E_MAX_ 899 s) and a Hill coefficient (N 0.66). The equilibration half time (T_1/2_keo) was 1.4 min. Results for the reaction time were plasma concentration (Cp_50_ 65.3 mg.L^−1^), E_MAX_ 34 min, N 0.80 with a baseline thromboelastometric reaction time of 0.4 min. The equilibration half time (T_1/2_keo) was 2.04 min.

**Conclusions:**

Dabigatran reversibly binds to the active site on the thrombin molecule, preventing thrombin‐mediated activation of coagulation factors. The effect compartment model performed slightly better than the turnover model and was able to adequately capture pharmacodynamics for both activated clotting and thromboelastometric reaction times. The equilibration half time was short (<2 min). These data can be used to inform future animal preclinical studies for those undergoing cardiopulmonary bypass. These preclinical data also demonstrate the magnitude of parameter values for a delayed effect compartment model that are applicable to humans.

What is already known about this subject

Dabigatran is a possible alternative to heparin for children requiring cardiopulmonary bypass. A dabigatran concentration–coagulant response relationship has not been described in humans.

What this study adds

A concentration–response relationship for anticoagulant effect was described in New Zealand white rabbits using activated clotting time and thromboelastometric reaction time. These data can be used to inform both animal preclinical studies for those undergoing cardiopulmonary bypass and for quantification of effect in humans.

## 1 INTRODUCTION

Dabigatran etexilate is a direct thrombin inhibitor used for oral administration in humans for thromboprophylaxis and treatment. Preliminary results suggest that the effects of dabigatran concentration on diluted thrombin time (dTT), ecarin clotting time (ECT), and activated partial thromboplastin time (aPTT) are largely comparable between adults and children, except in those aged younger 2 months; developmental hemostatic changes had little impact on response to dabigatran.^1^ The U.S. Food and Drug Administration (FDA) has recently approved dabigatran oral pellets for the treatment of children ages 3 months–12 years with venous thromboembolism (VTE) directly after receiving injectable anticoagulants for at least 5 days. The treatment also is approved as prophylaxis for recurrent thrombosis in patients ages 3 months–12 years who have completed treatment for first VTE.^2^

Other direct thrombin inhibitors (e.g., bivalirudin, hirudin, argatropan) have been successfully used for patients undergoing cardiopulmonary bypass. Bivalirudin has been used for children undergoing cardiopulmonary bypass with anti‐thrombin 3 deficiency and heparin resistance, heparin‐induced thrombocytopenia with vascular thrombosis (HIT), and for those who have suffered anaphylaxis to protamine.^3^, ^4^ Bivalirudin dose, however, remains uncertain for children undergoing cardiopulmonary bypass.^5^ Dabigatran is an alternative drug that has promise as a direct thrombin inhibitor for cardiopulmonary bypass ^6^, ^7^ because the effects of dabigatran can be rapidly and completely reversed with the monoclonal antibody idarucizumab.^8^

Further pediatric clinical trials assessing the relationship between coagulation assay responses and clinical outcome are needed to confirm coagulation response similarities between children and adults.^1^ Reports of concentration–response relationships are lacking in children. Investigation using animal models is a first step used to guide investigation in humans. This response relationship was investigated in rabbits using both an effect compartment model ^9^, ^10^ and an indirect response (turnover) model.^11^ The effect measures of reaction time (R) and activated clotting time (ACT) were used as convenient point‐of‐care whole blood tests reflecting inhibition of the tissue factor pathway and contact activation, respectively. The ACT is commonly used clinically to assess the adequacy of high‐dose heparin anticoagulation for cardiopulmonary bypass and has also been used when bivalirudin is administered for this indication.^12^ The NZ white rabbit was used for investigation because it has similar size and renal function to a neonate of 50 weeks postmenstrual age.

## 2 METHODS

### 2.1 Animals and materials

The 10 New Zealand White rabbits (4–4.5 kg) used for study were maintained in a pathogen‐free vivarium under recommendations of the National Institutes of Health Guide for the Care and Use of Laboratory Animals, with a 12:12‐h, light–dark cycle and food and water available ad libitum. All experimental protocols were approved by the Association for Assessment and Accreditation of Laboratory Animal Care‐accredited University of Rochester Committee on Animal Resources.

The central ear artery and a marginal ear vein were aseptically prepared in 10 rabbits and cannulated with 24 g IV catheters (Jelco^®^, ICU Medical, Inc.). Rabbits were restrained and dabigatran (15 mg.kg^−1^) was injected through the venous cannula manually over 15 sec. Samples (2 ml) were collected into citrate tubes from the arterial cannula at baseline (prior to injection) and at 5, 15, 30, and 60 min while the rabbits were restrained. Rabbits were then moved back to their cages with food and water ad libitum. The remaining blood samples at 120, 180, 300, and 420 min were collected without restraining. The total amount of collected blood was less than 8 ml.kg^−1^. Rabbits were monitored for 24 h for adverse reactions.

### 2.2 Dabigatran solutions

One milligram of Dabigatran (Clearsynth, Ontario, Canada) was dissolved in 60 μl of 0.075 M HCl, and then the solution was added to 0.2 ml of 20% N,N‐dimethylacetamide. Based on this recipe in vivo injection of 15 mg.kg^−1^ Dabigatran into 4 kg rabbit required 60 mg of Dabigatran dissolved in 3.6 ml of 0.075 M HCl, and diluted in 12 ml of 20% N,N‐dimethylacetamide, with a total injection volume of 15.6 ml.

### 2.3 Rapid TEG, ACT+, Hb, and LC–MS/MS

Reaction time (R), activated clotting time (ACT), and dabigatran concentration were measured in all blood samples. Kaolin/tissue‐factor‐activated thromboelastography (rTEG) measurements were performed using a Thromboelastograph Analyzer 5000 (Haemoscope Corp.). The reaction was initiated when 340 μl of blood was mixed with 10 μl of rTEG reagents and 20 μl of 0.2 M CaCl_2_ was added to recalcify the citrated specimen. The reaction was monitored until reaction time (R) was established. Activated clotting time (ACT) was measured using the Hemochron Signature Elite (Accriva Diagnostics, Inc.) according to the manufacturer's instructions. One milliliter of blood was centrifuged at 370 *g* for 15 min to obtain plasma dabigatran concentrations measured using liquid chromatography/mass spectrometry (LC–MS/MS) with a Dionex Ultimate 3000 UHPLC coupled to a Q Exactive Plus mass spectrometer (Thermo Scientific).

### 2.4 Dabigatran Pharmacokinetic–Pharmacodynamics

Population parameter estimates were obtained using nonlinear mixed effects models (NONMEM 7.4, ICON Development Solutions). This model accounts for population parameter variability (between subjects) and residual variability (random effects) as well as parameter differences predicted by covariate (fixed) effects. Population parameter variability was described using exponential models, which is equivalent to assuming a log‐normal distribution and avoids biologically inappropriate parameter values of zero or less. Residual unidentified variability (RUV) was modeled using both proportional (RUV_PROP_) and additive residual (RUV_ADD_) errors. The ADVAN6 subroutine was used to solve differential Equations. NM‐TRAN code is available in supplementary material (**Supplementary NM‐TRAN Codes**). A sequential PPPD method was used for final pharmacodynamic parameter estimates.^13^ Convergence criterion was three significant digits.

#### 2.4.1 Pharmacokinetics

A two‐compartment (central and peripheral) pharmacokinetic model was used to fit data. The model was parameterized in terms of clearance (CL), between compartment clearance (Q), central volume (V1), and peripheral volume of distribution (V2). The pharmacokinetic parameter values were standardized for a body weight of 70 kg using allometric models.^14^ This standardization allows comparison of rabbit parameter estimates with those reported for human adults ^15^:

Pi=PSTD×WiWSTDEXP

where P_i_ is the parameter of the i^th^ subject, W_i_ is the weight of the i^th^ subject, and P_STD_ is the parameter of standard weight W_STD_ of 70 kg. The EXP exponent was 0.75 for clearance and 1 for distribution volumes.^16^

#### 2.4.2 Pharmacodynamics

A sigmoidal E_MAX_ model was used to describe both activated clotting time (ACT) and thromboelastogram reaction time (R). Population parameter estimates were estimated using an effect compartment model, a model valid for situations where there is an apparent temporal displacement between plasma concentration (Cp) and response, for example, neuromuscular blocking drugs.^9^ A rate constant (keo, T_1/2_keo = Ln(2)/keo) links plasma concentration with effect site concentration (Ce).

Effect=E0+EMAXxCeNCe50N+CeN

The parameter E0 is the baseline measure (e.g., ACT 100, R 0.4 min), E_MAX_ is the maximum drug effect, Ce_50_ is the effect site concentration eliciting half of E_MAX_ and N is the Hill coefficient describing the steepness of the concentration–response curve.^15^

**FIGURE 1:**
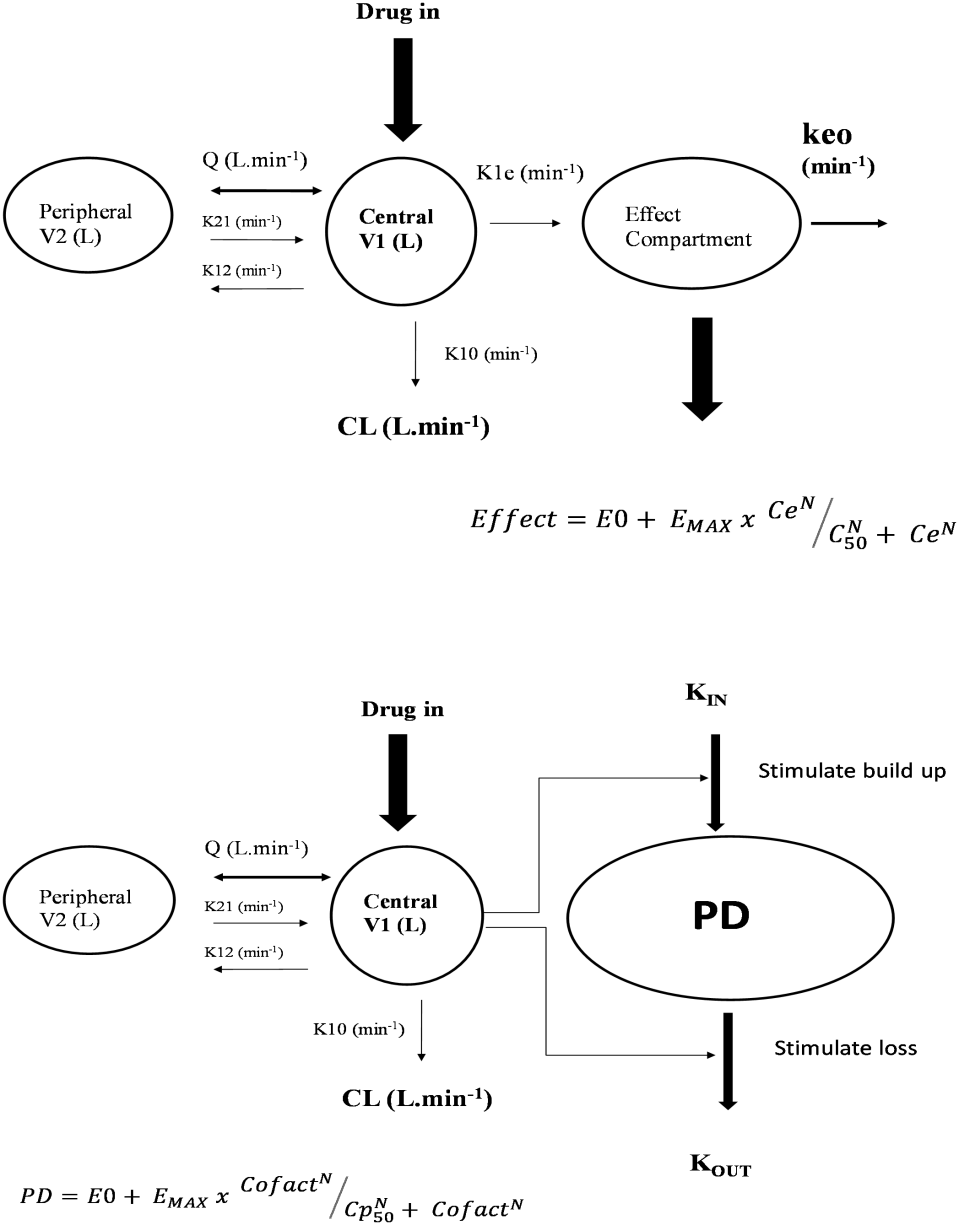
A diagram representing the two models. The upper panel demonstrates the effect compartment model. Drug is delivered into a central compartment (V1) that distributes to a peripheral compartment (V2) linked by an intercompartment clearance (Q). A rate constant (k1e) links the central compartment to an effect compartment. The rate constant k1e is the same as keo when the system is at equilibrium. The lower panel demonstrates an indirect response (turnover) model. This model assumes that pharmacodynamic effect (i.e., PD, inhibition or stimulation) are due to factors (Cofact) that control the production or dissipation of drug response The measured response (PD) to dabigatran is due to factors controlling the synthesis turnover rate, kin (TNOVER = Ln (2)/kin).

An indirect effect model was also investigated. Indirect effect (turnover) models assume that pharmacodynamic effect (i.e., PD, inhibition, or stimulation) are due to factors (Cofact) that control the production or dissipation of drug response, for example, warfarin effect mediated through prothrombin complex.^17^ The measured response (PD) to dabigatran is due to factors controlling the synthesis turnover rate, kin (TNOVER = Ln (2)/kin) (Figure 1)

PD=E0+EMAXxCofactNCp50N+CofactN

#### 2.4.3 Quality of fit

Model selection required an improvement in the NONMEM objective function (OBJ) between nested models, equating to a reduction >3.84 based on a Chi square distribution (*α* < 0.05). A visual predictive check (VPC), was used to evaluate how well the model predicted the distribution of observed dabigatran concentrations or coagulation measures (ACT and R).

## 3 RESULTS

Pharmacokinetic population parameter estimates for a body weight of 70 kg are shown in Table 1. Parameter estimates are scaled to a typical person of 70 kg for convenience of comparing characteristics between species. This does not change the relationship between size and parameters; it simply changes the scale of the parameter. ^15^ The visual predictive check (VPC) is shown in Figure S1.

**TABLE 1:** Standardized dabigatran population pharmacokinetic parameter estimates

Parameter	Estimate	BSV%	SE%	95% CI
CL_std_ (L.min^−1^.70 kg^−1^)	0.135	24.7	8.0	0.109–0.157
V1_std_ (L.70 kg^−1^)	12.3	12.3	8.5	11.4–12.4
Q_std_ (L.min^−1^.70 kg^−1^)	0.33	7.8	20.5	0.291–0.349
V2_std_ (L.70 kg^−1^)	30.1	10	40.2	25.9–38.7
RUV_ADD_ (mg/L)	1.31	‐	11.2	0.05–1.48
RUV_PROP_ (%)	0.9	‐	81.9	0.02–9

*Note*: BSV is the between subject parameter variability, SE is the standard error of the structural parameter, and CI is the confidence interval.

**FIGURE 2:**
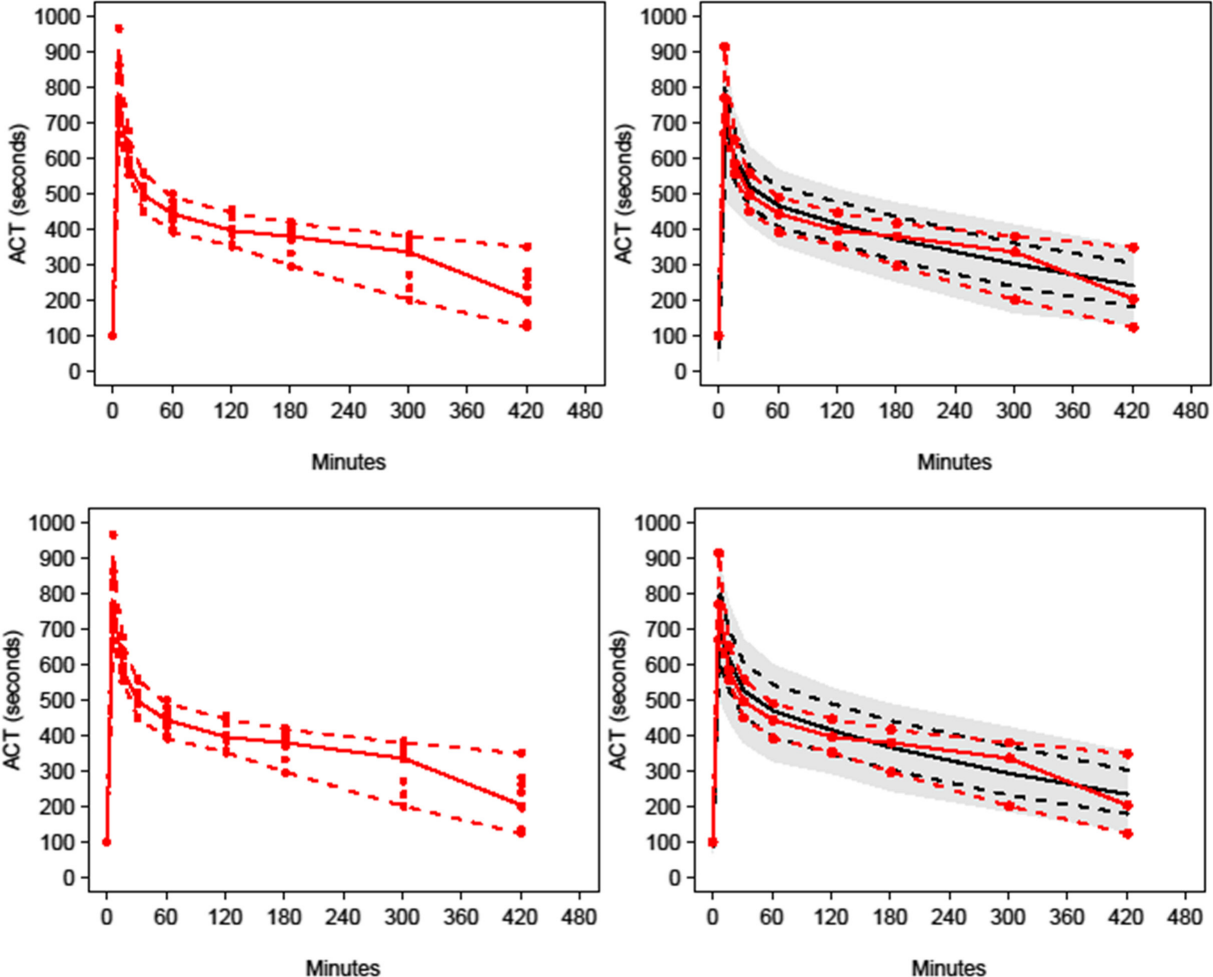
Population prediction corrected visual predictive checks (PC‐VPC) for the pharmacodynamic activated clotting time response. All plots show median and 90% confidence intervals (solid and dashed lines). Left‐hand plot shows all observed concentrations. Right‐hand plot shows percentiles (10, 50, 90) for observations (lines with symbols) and predictions (lines) with 95% confidence intervals for prediction percentiles (gray‐shaded areas). The upper panel shows that for an effect compartment model while the lower is for a turnover model.

**FIGURE 3:**
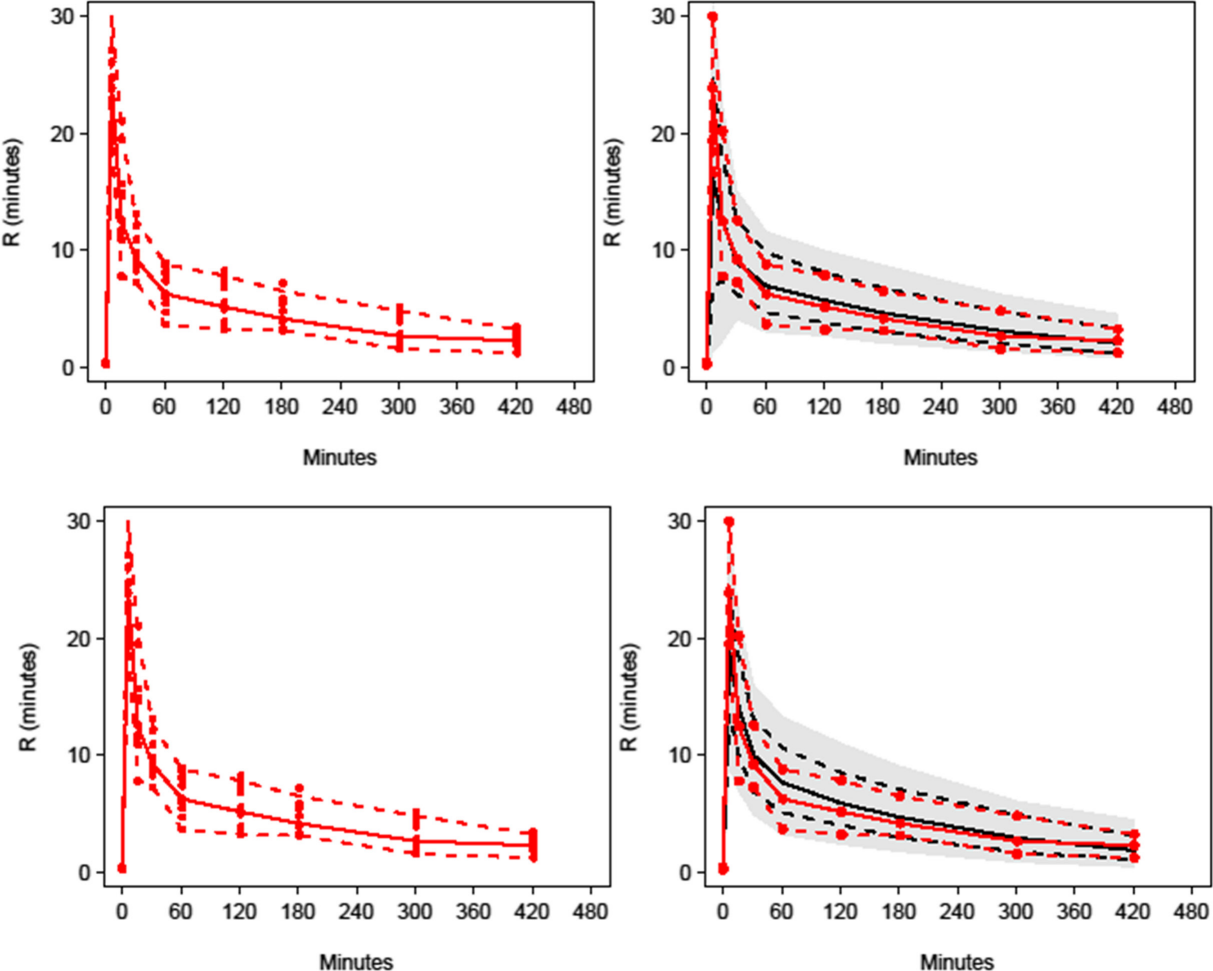
Population prediction corrected visual predictive checks (PC‐VPC) for the pharmacodynamic reaction time (R) response. The upper panel shows that for an effect compartment model while the lower is for a turnover model. The effect compartment model shows adequacy of model predictions with little apparent deviations between model and data. Reaction time at approximately 1 h shows some deviation between model and data in the turnover model.

Pharmacodynamic population parameter estimates for activated clotting times are shown in Table 2 and for reaction time in Table 3. The visual predictive check (VPC) plots for both the effect of dabigatran on activated clotting time (Figure 2) and reaction time (Figure 3) confirmed the adequacy of model predictions with little apparent deviations between model and data except for reaction time with use of the turnover model (Figure 3). The 90% confidence interval and median for observed data lies within the predicted intervals were obtained by simulation.

**TABLE 2:** Pharmacodynamic population parameter estimates for activated clotting times (ACT)

Parameter	Estimate	BSV%	95% CI	Estimate	BSV%
Effect compartment model				Turnover model	
E0 (sec)	100 FIX	‐	‐	100 FIX	‐
Emax (sec)	899 FIX	‐	‐	899 FIX	‐
Ce_50_ / Cp_50_ mg.L^−1^	20.1	0.7	19.7,22.6	25.9	26.2
*N*	0.66	‐	0.58, 0.77	0.671	‐
T_1/2_keo / TRNOVR (min)	1.4	16.2	0.74, 1.79	0.952	12.2
RUV_ADD_ (sec)	22.5	‐	16.0	0.025	‐
RUV_PROP_ (%)	135	‐	95, 169	8.5	‐

**TABLE 3:** Pharmacodynamic population parameter estimates for reaction times (R)

Parameter	Estimate	BSV%	95% CI	Estimate	BSV%
Effect compartment model				Turnover model	
E0 (min)	0.4 FIX	‐	‐	0.4 FIX	‐
Emax (min)	34 FIX	‐	‐	34 FIX	‐
Ce_50_ (mg/L)	65.3	0.7	51.7,83.9	47.6	26.2
*N*	0.80	‐	0.72, 0.86	0.917	‐
T_1/2_keo/TRNOVR (min)	2.04	16.2	1.28, 2.47	1.38	12.2
RUV_ADD_ (min)	0.116	‐	0.06, 0.15	0.09	‐
RUV_PROP_ (%)	10.3	‐	8.7, 11.5	19.3	‐

## 4 DISCUSSION

Dabigatran was an effective anticoagulant. Although it is formulated as the etexilate mesylate salt for oral use, it was prepared in a solution appropriate for intravenous administration. The intravenous preparation allows characterization of pharmacokinetics without confounding variability influences such as relative bioavailability or absorption parameters. This intravenous formulation was well tolerated by the rabbits, without apparent sequelae despite high coagulation measures observed.

Dabigatran has a volume of distribution of 50–70 L in humans with an elimination half‐life of 12–17 h. The drug is commonly administered orally and relative bioavailability is only 3%–7%. Human clearance estimates (~4 L.min^−1^.70 kg^−1^) were half those estimated in rabbits (~8 L.min^−1^.70 kg^−1^).^18^, ^19^ However, clearance is influenced by renal function.^20^ and renal function, corrected for size using allometry, is 30–60 ml/min/70 kg in white NZ rabbits,^21^ half that in healthy adult humans, suggesting that clearance by hepatic glucuronide metabolism to acyl metabolotes has importance in rabbits. We estimated a volume of distribution (42 L.70 kg^−1^) that was similar to humans.^18^

The effects of dabigatran on coagulation were adequately described in rabbits. Both effect compartment and turnover models estimated similar pharmacodynamic parameter values. Dabigatran reversibly binds to the active site on the thrombin molecule, preventing thrombin‐mediated activation of coagulation factors. Unlike warfarin, drug activity is not mediated through secondary proteins dependent on synthesis or breakdown. Onset of effect was rapid and the delayed effect compartment model is more physiologically appropriate.

Delayed effect compartment models are commonly used to model depth of consciousness attributed to anesthesia drugs. The turnover model is more appropriate when the delay between drug concentration and effect is due to a lag in pharmacodynamic processes rather than equilibration of drug in an effect compartment (the biophase). The observed effect is part of a dynamic process where the baseline effect (e.g., E0 for ACT or R) may be a balance between the apparent rate of “production” of the effect and rate of “removal” of the effect (Figure 1). First‐order rate constants (kin and kout) represent multiple processes.^22^ While warfarin coagulation effects are best represented using a turnover model, the delayed effect compartment better represents effects due to dabigatran.

Dabigatran effect on coagulation has been described as direct when the drug is given orally. A pharmacodynamic model using the effect measures, activated partial thromboplastin time (aPTT) and ecarin clotting time (ECT), has been reported using a direct pharmacodynamic relationship after oral administration.^23^ This model is reasonable given the variability associated with oral absorption parameters, the short equilibration time (T_1/2_keo), and the concentration range investigated. The effects of inogatran, a synthetic low‐molecular‐weight thrombin inhibitor, have been described for aPPT using a direct relationship with combination linear and Emax models.^24^ Similar results are reported for melagatran.^25^ We report a short equilibration time for the effect models using reaction time and activated clotting time. This short equilibration time is unlikely to have clinical impact; it is small and testing of coagulation status is invariably undertaken 3–4 min after intravenous drug administration.

The current analysis involving animals is a common route toward further study in humans, both adults and children. Such models of hemostasis may be extrapolated to humans, but there are few studies that have compared coagulation and fibrinolysis across species.^26^ Preclinical studies in white New Zealand rabbits may not be ideal for extrapolation to humans because rabbit thrombin is may be more resistant to inhibition that human thrombin. Rabbit plasmin generation also differs from human.^26^ This current study does, however, inform future preclinical animal and human studies involving anticoagulation for cardiopulmonary bypass. It also demonstrates the magnitude of parameter values for a delayed effect compartment model applicable to humans.

## CONFLICTS OF INTEREST

Michael Eaton is a holder of Provisional Patent Application No. 62/814454. Anticoagulant Compositions and Uses Thereof. Brian Anderson is Associate Editor in Chief for Pediatric Anesthesia.

## ETHICAL APPROVAL

All experimental protocols were approved by the Association for Assessment and Accreditation of Laboratory Animal Care‐accredited University of Rochester Committee on Animal Resources.

## Supporting information


Figure S1

**Appendix S1** Supporting Information

## Data Availability

The data that support the findings of this study are available from the corresponding author upon reasonable request.
