# Supplementary figures and images for: Delayed concentration effect models for dabigatran anticoagulation
Source: Paediatr Anaesth. 2022 Jul 2;32(10):1113–20. doi: 10.1111/pan.14511 (PMC9541555; doi:10.1111/pan.14511)

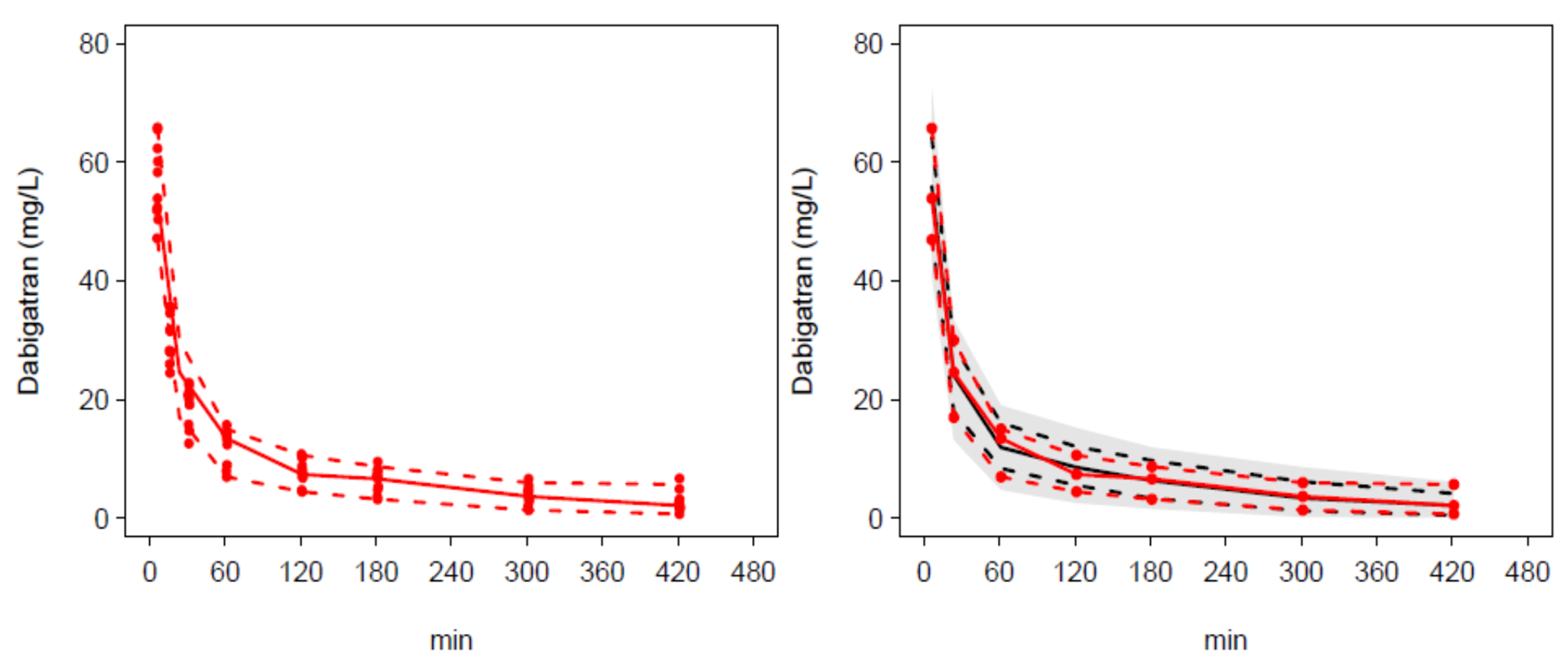

Supplement: Supplementary file 1 — Figure S1 [file PAN-32-1113-s002.tif]
