# Supplementary material for: Delayed concentration effect models for dabigatran anticoagulation
Source: Paediatr Anaesth. 2022 Jul 2;32(10):1113–20. doi: 10.1111/pan.14511 (PMC9541555; doi:10.1111/pan.14511)
Supplement: Supplementary file 2 — Appendix S1 Supporting Information [file PAN-32-1113-s001.docx]

Supplementary Material

# NM-TRAN Code for Effect Compartment Model

$PROB dabigatran effect compartment model using R time

$INPUT IDX ID TIMX TIME MDV CMT DUR RATE AMT DVID DV WT

$DATA Rabbit_dabiPD.csv IGNORE #

$ESTIM MAXEVAL=9999 NSIG=3 SIGL=9 PRINT=1 NOABORT METHOD=CONDITIONAL INTERACTION

MSFO=dabi_2cmt_PKPD.msf

$COV

$THETA (0.01,0.135,200) FIX ; CL

$THETA (0.5,12.3,200) FIX ; V1

$THETA (0.001,0.331,200) FIX ; Q

$THETA (0.5,30.,200) FIX ; V2

;RESIDUAL UNIDENTIFIED VARIABILITY

$THETA (0,1.32,) FIX ; RUV_SDCP

$THETA (0,0.5,) FIX ; RUV_CVCP

$OMEGA BLOCK(4)

0.0612 ; PPVCL

0.00863 0.016 ; PPVV1

0.00777 0.0093 0.0062 ;PPVQ

0.0165 -0.00188 0.0008 0.02 FIX ; PPVV2

; PD

$THETA (0.1,34, 50 ) FIX ; EMAX

$THETA (0.1,0.752,8) ; HILL

$THETA (0.001,39.6, ) ; C50

$THETA (0.1,0.4,1) FIX ; E0

$THETA (0.001,1.68,200) ; TEQ

;RESIDUAL UNIDENTIFIED VARIABILITY

$THETA (0,0.532,) ; RUV_SDPD

$THETA (0,0.0692,) ; RUV_CVPD

$OMEGA BLOCK (2)

0.5 ; PPVC50

0.1 0.5 ;PPVTEQ

$OMEGA 0 FIX ; PPVE0

$OMEGA 0 FIX ; PPVEMX

$OMEGA 0 FIX ; PPVHIL

;RESIDUAL UNIDENTIFIED VARIABILITY (OBSERVATIONS)

$OMEGA 0 FIX ; PPV_RUVCP

$OMEGA 0 FIX ; PPV_RUVPD

$SIGMA 1. FIX ; EPS1

$SUBR ADVAN6 TOL=5

$MODEL

COMP (DIGAB)

COMP (PERIPH)

COMP (EFFECT)

$PK

IF (AMT.GT.0) DOSE=AMT

IF (NEWIND.LE.1) THEN

DOSE=0

LN2=LOG(2)

ENDIF

FSZV=WT/70

FSZCL=FSZV**0.75

FSZT=FSZV**0.25

CL=FSZCL*CL*EXP(PPVCL)

Q=FSZCL*Q*EXP(PPVQ)

V1=FSZV*V1*EXP(PPVV1)

V2=FSZV*V2*EXP(PPVV2)

S1=V1

S2=V2

D1=DUR

E0=E0*EXP(PPVE0)

C50=C50*EXP(PPVC50)

EMAX=EMAX*EXP(PPVEMX)

HILL=HILL*EXP(PPVHIL)

TEQ=FSZT*TEQ*EXP(PPVTEQ)

KEQ=LN2/TEQ

$DES

DCP=A(1)/V1

DC2=A(2)/V2

DCE=A(3)

DADT(1)= -DCP*CL-DCP*Q +DC2*Q

DADT(2)= Q*DCP-DC2*Q

DADT(3)= KEQ*(DCP-DCE)

$ERROR

CP=A(1)/V1

CE=A(3)

"IF (CE.LE.0)CE=1D-10

CEN=CE**HILL

C50N=C50**HILL

FX=E0 + EMAX*CEN/(C50N+CEN)

PROPP=CP*RUV_CVCP

ADDP=RUV_SDCP

SDCP=SQRT((PROPP*PROPP) + (ADDP*ADDP))*EXP(PPV_RUVCP)

PROPD=CE*RUV_CVPD

ADDPD=RUV_SDPD

SDPD=SQRT((PROPD*PROPD) + (ADDPD*ADDPD))*EXP(PPV_RUVPD)

IF(DVID.LE.1) THEN ;; DIGABACTRAN CONCENTRATION

Y=CP + SDCP*EPS1

ENDIF

IF(DVID.EQ.2) THEN ;; EFFECT COAG

Y=FX + SDPD*EPS1

ENDIF

$TABLE ID TIME WT CL V1 Q V2 Y MDV DVID WT

ONEHEADER NOPRINT FILE=dabi_2cmt_PD_R.fit

# NM-TRAN Code for Turnover Model

$PROB dabigatran turnover model using R Time

$INPUT IDX ID TIMX TIME MDV CMT DUR RATE AMT DVID DV WT

$DATA Rabbit_dabiPD.csv IGNORE #

$ESTIM MAXEVAL=9999 NSIG=3 SIGL=9 PRINT=1 NOABORT METHOD=CONDITIONAL INTERACTION

MSFO=dabi_2cmt_PKPD.msf

$COV

$THETA (0.01,0.135,200) FIX ; CL

$THETA (0.5,12.3,200) FIX ; V1

$THETA (0.001,0.331,200) FIX ; Q

$THETA (0.5,30.,200) FIX ; V2

;RESIDUAL UNIDENTIFIED VARIABILITY

$THETA (0,1.32,) FIX ; RUV_SDCP

$THETA (0,0.5,) FIX ; RUV_CVCP

$OMEGA BLOCK(4)

0.0612 ; PPVCL

0.00863 0.016 ; PPVV1

0.00777 0.0093 0.0062 ;PPVQ

0.0165 -0.00188 0.0008 0.02 FIX ; PPVV2

; PD

$THETA (1,34., 40) FIX ; EMAX 34 fix as for delayed effects

$THETA (0.1,0.917,8) ; HILL

$THETA (10,47.6, 50 ) ; C50

$THETA (0.1 0.4,0.4) FIX ; E0

$THETA (0.001,1.38, 1000) ; TOVER

;RESIDUAL UNIDENTIFIED VARIABILITY

$THETA (0,0.091,) ; RUV_SDPD

$THETA (0,0.193,) ; RUV_CVPD

$OMEGA BLOCK (2)

0.0122 ; PPVC50

-0.00595 0.155 ; PPVTVR

$OMEGA 0 FIX ;PPVEMX

$OMEGA 0 FIX ; PPVE0

;RESIDUAL UNIDENTIFIED VARIABILITY (OBSERVATIONS)

$OMEGA 0 FIX ; PPV_RUVCP

$OMEGA 0 FIX ; PPV_RUVPD

$SIGMA 1. FIX ; EPS1

$SUBR ADVAN6 TOL=5

$MODEL

COMP (DIGAB)

COMP (PERIPH)

COMP (TRNOVR)

$PK

IF (AMT.GT.0) DOSE=AMT

IF (NEWIND.LE.1) THEN

DOSE=0

LN2=LOG(2)

ENDIF

FSZV=WT/70

FSZCL=FSZV**0.75

FSZT=FSZV**0.25

CL=FSZCL*CL*EXP(PPVCL)

Q=FSZCL*Q*EXP(PPVQ)

V1=FSZV*V1*EXP(PPVV1)

V2=FSZV*V2*EXP(PPVV2)

S1=V1

S2=V2

D1=DUR

E0=E0*EXP(PPVE0)

C50=C50*EXP(PPVC50)

EMAX=EMAX*EXP(PPVEMX)

HILL=HILL

;TOVER is turnover half-life

TOVER=FSZT*TOVER*EXP(PPVTVR)

A_0(3)=E0

$DES

DCP=A(1)/V1

DC2=A(2)/V2

DCOF=A(3)

KOUT=LN2/TOVER

RIN=KOUT

C50N=C50**HILL

DCOFN=DCOF**HILL

PD=E0 + EMAX*DCOFN/(C50N+DCOFN)

DADT(1)= DC2*Q-DCP*(CL+Q)

DADT(2)= Q*(DCP-DC2)

DADT(3)= RIN*PD -KOUT*DCOF

$ERROR

CP=A(1)/V1

COF=A(3)

PROPP=CP*RUV_CVCP

ADDP=RUV_SDCP

SDCP=SQRT((PROPP*PROPP) + (ADDP*ADDP))*EXP(PPV_RUVCP)

PROPD=COF*RUV_CVPD

ADDPD=RUV_SDPD

SDPD=SQRT((PROPD*PROPD) + (ADDPD*ADDPD))*EXP(PPV_RUVPD)

IF(DVID.LE.1) THEN ;; DIGABACTRAN CONCENTRATION

Y=CP + SDCP*EPS1

ENDIF

IF(DVID.EQ.2) THEN ;; EFFECT COAG

Y=COF + SDPD*EPS1

ENDIF

$TABLE ID TIME WT CL V1 Q V2 Y MDV DVID WT

ONEHEADER NOPRINT FILE=dabi_2cmt_trnovrR.fit
